# Supplementary material for: Updated Trends on the Biodiscovery of New Marine Natural Products from Invertebrates
Source: Mar Drugs. 2022 Jun 9;20(6):389. doi: 10.3390/md20060389 (PMC9228037; doi:10.3390/md20060389)
Supplement: Supplementary file 1 [file marinedrugs-20-00389-s001.zip › marinedrugs-1761589-supplementary.pdf]

Updated trends on the biodiscovery of new marine natural products from invertebrates

Ricardo Calado, Renato Mamede, Sónia Cruz and Miguel C. Leal

Supplementary Material

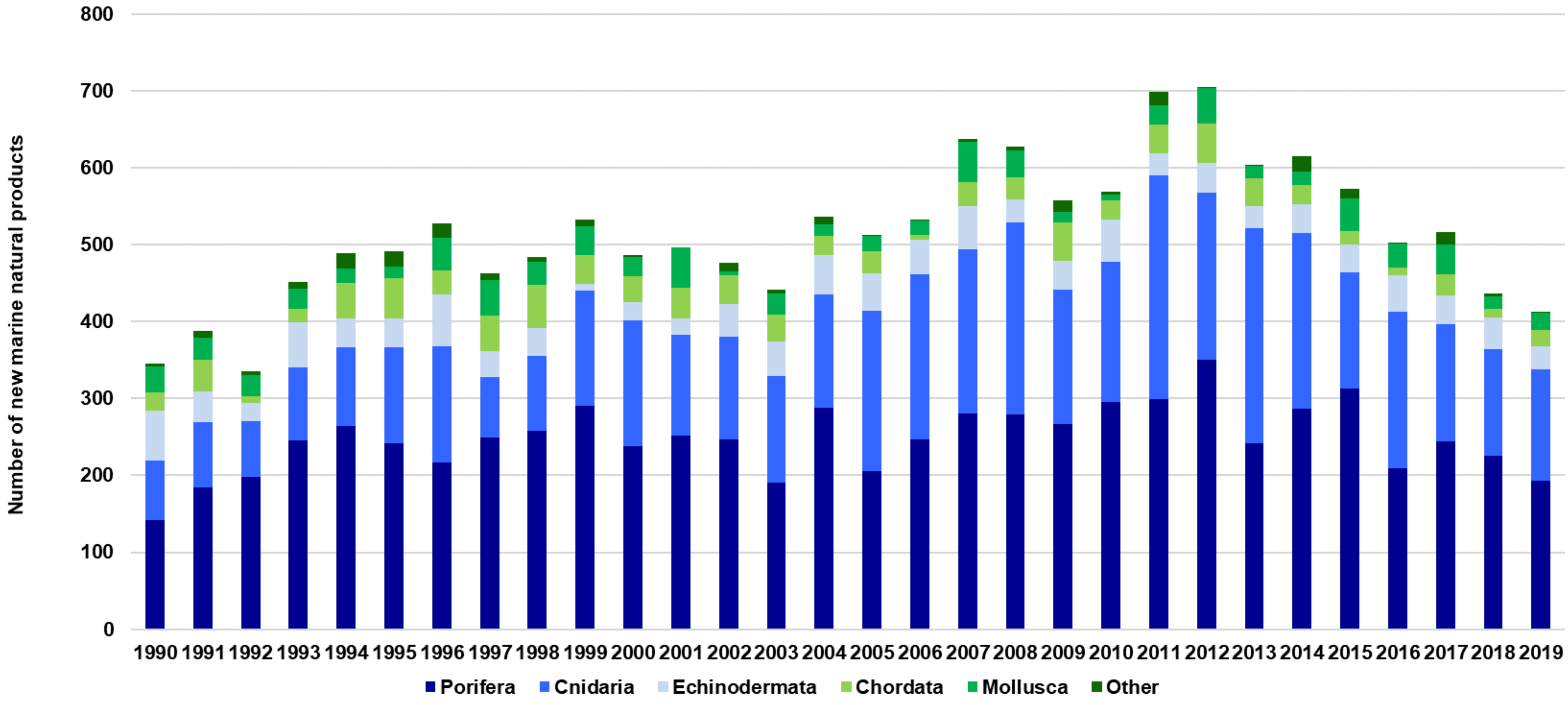

Figure S1: Number of new marine natural products from invertebrates discovered between 1990 and 2019 (Chordata solely refers to tunicates; “Other” refer to phyla Annelida, Bryozoa, Arthropoda, Brachiopoda, Hemichordata and Platyhelmintes).
